# Risk of COVID-19 infection, hospitalization and mortality in psoriasis patients treated with interleukin-17 inhibitors: A systematic review and meta-analysis

**DOI:** 10.3389/fimmu.2022.1046352

**Published:** 2022-10-21

**Authors:** Meitong Liu, Huijuan Wang, Lu Liu, Saijin Cui, Xiangran Huo, Zhuoyun Xiao, Yaning Zhao, Bin Wang, Guoqiang Zhang, Na Wang

**Affiliations:** ^1^ Cancer Institute, The Fourth Hospital of Hebei Medical University, Shijiazhuang, China; ^2^ Department of Dermatology, The First Hospital of Hebei Medical University, Shijiazhuang, China; ^3^ Candidate Branch of National Clinical Research Center for Skin Diseases, Shijiazhuang, China

**Keywords:** COVID-19, interleukin-17 inhibitors, psoriasis, NNT, meta-analysis, systematic review

## Abstract

**Background:**

Coronavirus disease 2019 (COVID-19) have brought great disaster to mankind, and there is currently no globally recognized specific drug or treatment. Severe COVID-19 may trigger a cytokine storm, manifested by increased levels of cytokines including interleukin-17 (IL-17), so a new strategy to treat COVID-19 may be to use existing IL-17 inhibitors, which have demonstrated efficacy, safety and tolerability in the treatment of psoriasis. However, the use of IL-17 inhibitors in patients with psoriasis during the COVID-19 pandemic remains controversial due to reports that IL-17 inhibitors may increase the risk of respiratory tract infections.

**Objectives:**

The systematic review and meta-analysis aimed to evaluate the effect of IL-17 inhibitors on the risk of COVID-19 infection, hospitalization, and mortality in patients with psoriasis.

**Methods:**

Databases (including Embase, PubMed, SCI-Web of Science, Scopus, CNKI, and the Cochrane Library) were searched up to August 23, 2022, for studies exploring differences in COVID-19 outcomes between psoriasis patients using IL-17 inhibitors and those using non-biologics. Two authors independently extracted data and assessed the risk of bias in a double-blind manner. The risk ratios (RRs) and 95% confidence intervals (CIs) were calculated and heterogeneities were determined by the *Q* test and *I* ^2^ statistic. And the numbers needed to treat (NNTs) were calculated to assess the clinical value of IL-17 inhibitors in preventing SARS-CoV-2 infection and treating COVID-19.

**Results:**

Nine observational studies involving 7,106 participants were included. The pooled effect showed no significant differences in the rates of SARS-CoV-2 infection (*P* = 0.94; *I* ^2^ = 19.5%), COVID-19 hospitalization (*P* = 0.64; *I* ^2^ = 0.0%), and COVID-19 mortality (*P* = 0.32; *I* ^2^ = 0.0%) in psoriasis patients using IL-17 inhibitors compared with using non-biologics. Subgroup analyses grouped by age and COVID-19 cases, respectively, revealed consistent results as above. Meanwhile, the pooled NNTs showed no significant differences between the two groups in the clinical value of preventing SARS-CoV-2 infection and treating COVID-19.

**Conclusion:**

The use of IL-17 inhibitors in patients with psoriasis does not increase the risk of SARS-CoV-2 infection or worsen the course of COVID-19.

**Systematic review registration:**

https://www.crd.york.ac.uk/prospero/, identifier CRD42022335195.

## Introduction

As of September 7, 2022, coronavirus disease 2019 (COVID-19) have caused more than 603 million confirmed cases, including more than 6.48 million deaths, and have brought great disaster to mankind (1). COVID-19 is a clinical syndrome caused by severe acute respiratory syndrome coronavirus 2 (SARS-CoV-2) for which there is currently no globally recognized specific drug or treatment. Severe COVID-19 may trigger a cytokine storm, a hyperinflammatory state triggered by viral infections, which primarily causes pneumonia followed by systemic inflammation (2–4). Among the variety of cytokines involved in the storm, interleukin-17 (IL-17) is a predominant mediator of pulmonary inflammation (5). Recently, several studies have shown that the levels of circulating IL-17 are elevated in the peripheral blood of COVID-19 patients (2, 6–8), so the use of IL-17 inhibitors may become a new treatment option for COVID-19, which directly block the IL-17 pathway (9).

As biologics, IL-17 inhibitors have been approved for the treatment of moderate-to-severe plaque psoriasis, and have demonstrated efficacy, safety and tolerability (10–14). However, IL-17 inhibitors have been reported to increase the risk of respiratory infections (15–17), so it needs to be considered whether psoriasis patients treated with IL-17 inhibitors are more susceptible to the virus during the COVID-19 pandemic. In addition, it is still unclear whether the use of IL-17 inhibitors can effectively treat COVID-19 or shorten the course of COVID-19. Although studies have compared the risk of SARS-CoV-2 infection and the course of COVID-19 in psoriasis patients receiving biologics (including IL-17 inhibitors) and those receiving non-biologics, the results are not entirely consistent. Therefore, we use a systematic review to explore the risk of COVID-19 infection, hospitalization, and mortality in psoriasis patients treated with IL-17 inhibitors during the COVID-19 pandemic.

## Materials and methods

### Study registration

This systematic review chose psoriasis patients using IL-17 inhibitors as the exposure group and psoriasis patients using non-biologics as the control group. And it was conducted according to the Preferred Reporting Items for Systematic reviews and Meta-Analyses (PRISMA) statement (18). The research protocol was registered in the PROSPERO database (CRD42022335195).

### Search strategy

To find studies exploring differences in COVID-19 outcomes between psoriasis patients using IL-17 inhibitors and those using non-biologics, we searched databases (including Embase, PubMed, SCI-Web of Science, Scopus, CNKI, and the Cochrane Library) for articles published in any language and gray literature before August 23, 2022, and manually searched the references from potentially relevant papers. The studies were screened by two reviewers (ML and LL) independently using Endnote, version X9 (Clarivate Analytics), and conflicts were resolved by a third reviewer (NW). For studies that lacked original data or unpublished, we would contact the authors by email.

MeSH (Medical Subject Headings) and free text terms for IL-17 inhibitors and COVID-19 were used, and the terms are as follows: (“COVID-19” OR “SARS-CoV-2” OR “coronavirus disease 2019” OR “severe acute respiratory syndrome coronavirus 2”) AND (“Psoriasis” OR “Psoriases”) AND (“Interleukin-17” OR “Secukinumab” OR “Ixekizumab” OR “Brodalumab” OR “Cosentyx” OR “Taltz” OR “Siliq” OR “Kyntheum” OR “Lumicef”). **Supplementary Table 1 (Table S1)** summarized the specific retrieval strategies and results used for each database.

### Inclusion and exclusion criteria

Original studies were included if they met all of the following criteria: (1) study design: observational studies or experimental (randomized controlled trials) studies; (2) population/participants: patients with psoriasis; (3) intervention/exposure: using IL-17 inhibitors; (4) control/comparison: using non-biologics; (5) outcomes: clear indicators of COVID-19 infection, hospitalization or mortality.

Studies were excluded if they met any of the following criteria: (1) no accessible clinical data or incomplete data; (2) duplicate published studies; (3) full-text not available.

Three endpoints of the review were: (1) presence of COVID-19 cases (including confirmed cases, defined as those with positive molecular tests, and suspected cases, defined as those who have possible COVID-19 symptoms); (2) hospitalization for COVID-19; (3) death from COVID-19.

### Definition of COVID-19 outcomes

The equations for SARS-CoV-2 infection rate (IR), COVID-19 hospitalization rate (HR), and COVID-19 mortality rate (MR) were as follows:


$$\;IR=\frac{I}{R}$$

$$HR=\frac{H}{R}$$

$$MR=\frac{M}{R}$$

where *I* is the number of COVID-19 cases, *R* is the number of people at risk of SARS-CoV-2 infection, *H* is the number of those hospitalized with COVID-19, and *M* is the number of those died from COVID-19.

### Data extraction and risk of bias assessment

The following detailed baseline characteristics were extracted from the included studies: study, type, country/region, period, age, type of IL-17 inhibitors, type of non-biologics, COVID-19 cases, number of exposure, number of control, and outcome indicator.

Two authors independently extracted data and assessed the risk of bias in a double-blind manner (ML and SC). Since the included studies were observational studies, the Strengthening the Reporting of Observational studies in Epidemiology (STROBE) statement was used (19). The STROBE statement allows for quality assessment of 22 items. The risk of bias rating for each item was reported as low, moderate, or high, and any discrepancies in data and rating were addressed by negotiation between the two or with a third author (HW).

### Statistical analysis

All statistical analyses were conducted with Stata, version 15 (StataCorp LLC) and RevMan, version 5.2 (The Nordic Cochrane Centre). All figures were generated by Stata and RevMan software, and processed by Adobe Illustrator, version CC 2018 (Adobe Systems Incorporated). The outcome measures were the risk ratios (RRs) of COVID-19 infection, hospitalization or mortality in patients with psoriasis receiving IL-17 inhibitors versus non-biologics. And the pooled numbers needed to treat (NNTs) and 95% confidence intervals (CIs) were calculated to estimate the absolute benefit or risk of using IL-17 inhibitors in preventing SARS-CoV-2 infection and treating COVID-19.

We evaluated statistical heterogeneity among studies using the *X* ^2^-based *Q* test and the *I* ^2^ statistic. The fixed-effect model with Mantel-Haenszel method was used when *P* > 0.05 of the *Q* test and *I* ^2^< 50%, otherwise the random-effect model with Inverse-Variance method was used, and then generated the pooled RRs and 95% CIs for all included studies. When no events were observed in one or both groups in an individual study, we used the fixed 0.5 correction value.

The NNT was calculated using the equation:


$$NNT=\frac{1}{CER-EER}$$

where *CER* is the control event rate and *EER* is the experimental event rate. We used the metannt command of the Stata software to calculate the NNTs. Metannt calculates NNT by deriving an effect size, applying it to a population with a given event prevalence, and from this deriving a projected event rate if the population were to receive the intervention. In this meta-analysis, we calculated NNT by using rates of SARS-CoV-2 infection, COVID-19 hospitalization, and COVID-19 mortality in the psoriasis patients using non-biologics (control event rate), RR (the effect size) and 95% CIs.

We conducted subgroup analyses grouped by age and COVID-19 cases, respectively. Age was divided into three levels: children (age<18 years), adults (age≥18 years), and NA (not available). COVID-19 cases were divided into two levels: confirmed cases and suspected cases. Also, we performed comparative analyses with IL-17 inhibitors according to the specific types of non-biologics, respectively. We undertook sensitivity analyses for the outcome indicators, which to account for potential study limitations that could lead to spurious precision of pooled effect estimates. We generated the funnel plots to explore the possibility of small study effects, and further assessed publication bias with Egger’s and Begg’s tests. If the *P* value of the test was less than 0.10, there was a publication bias.

To ensure that the population was not counted several times, we would take the following measures: if the exposure population was included in multiple studies, we would appropriately include only one of them; if several studies used the same control population, we would combine the exposure populations of these studies after eliminating duplicates (if any).

## Results

### Characteristics of included studies

**Figure 1:**
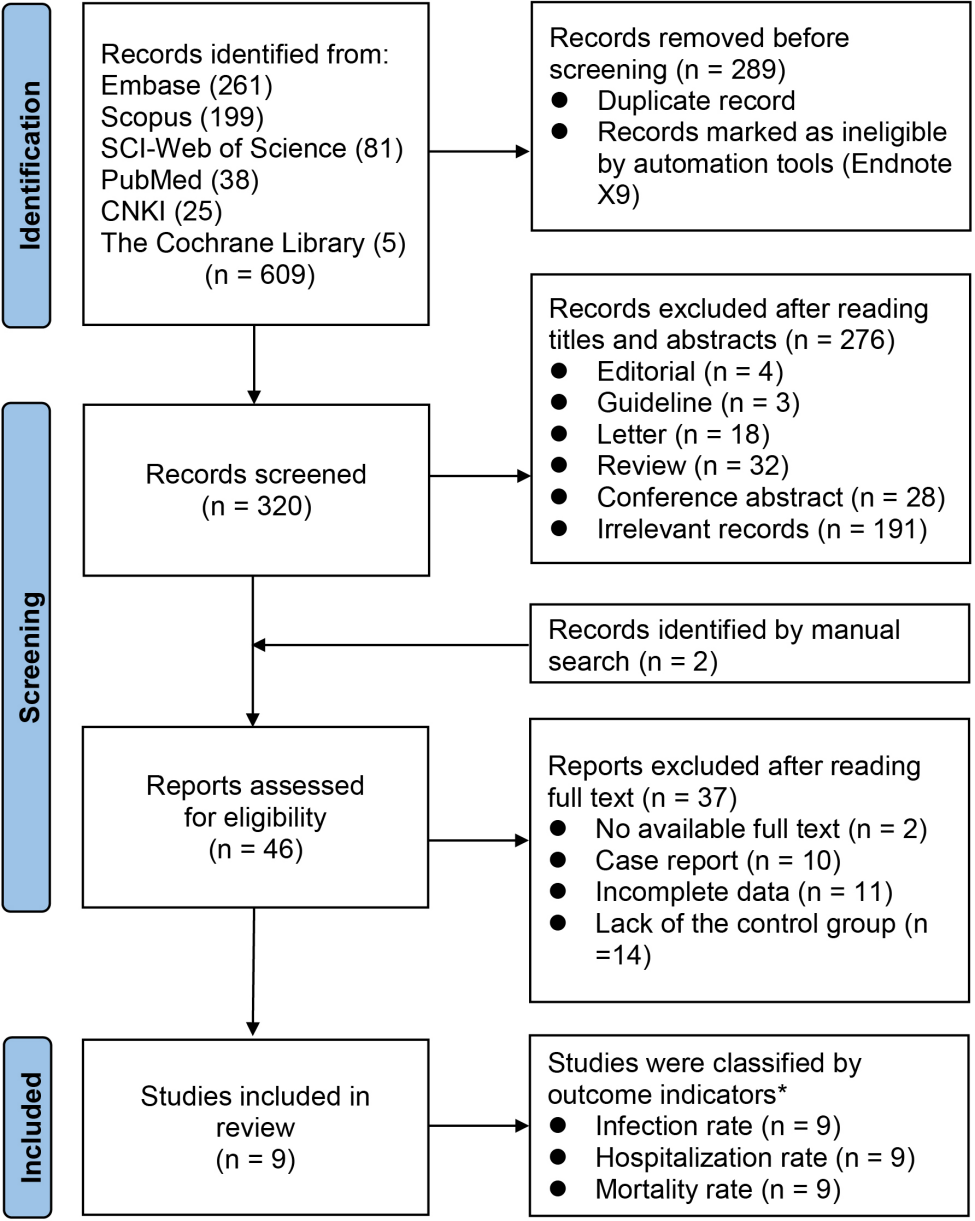
PRISMA flow chart for study selection. *n*, number; *PRISMA*, the Preferred Reporting Items for Systematic reviews and Meta-Analyses statement. ^*^All included studies contain three indicators.

We finally included 9 studies (20–28) involving 2,437 exposure individuals and 4,669 control individuals by databases and manual search in the meta-analysis (**Figure 1**), and we summarized the characteristics of all included studies (**Table 1**).

**Table 1:** Baseline Characteristics of Studies Included in the Meta-Analysis.

Study	Type	Country/Region	Period (MM/DD/YY)	Age	Type of IL-17 inhibitors	Type of non-biologics	COVID-19 cases	No. of exposure^a^		No. of control^b^		Outcome indicator(of COVID-19)
								Event	Total	Event	Total	
Beytout et al. (2021) (20)	multicenter, retrospective, survey	France	6/10/20-6/29/20	children	Secukinumab, Ixekizumab and Brodalumab	Topical therapies, Acitretin and Methotrexate	suspected cases	0	1	1	76	Infection rate^c^
								0	1	0	76	Hospitalization rate^c^
								0	1	0	76	Mortality rate^c^
Brazzelli et al. (2020) (21)	single-center, retrospective and prospective, cross-sectional	Italy	1/01/20-5/31/20	adults	NA	Topical therapies	suspected cases	5	21	17	100	Infection rate
								0	21	0	100	Hospitalization rate^c^
								0	21	0	100	Mortality rate^c^
Damiani et al. (2020) (22)	single-center, retrospective, case-control	Lombardy	2/21/20-4/09/20	adults	Secukinumab, Ixekizumab and Brodalumab	Apremilast and Dimetil-fumarate	confirmed cases	7	542	0	89	Infection rate^c^
								2	542	0	89	Hospitalization rate^c^
								0	542	0	89	Mortality rate^c^
Fougerousse et al. (2020) (23)	multicenter, retrospective, cross-sectional	France	4/27/20-5/07/20	adults	Secukinumab, Ixekizumab and Brodalumab	Methotrexate, Cyclosporine, Acitretin and Apremilast	suspected and confirmed cases	14	363	23	378	Infection rate^d^
								0	363	1	378	Hospitalization rate^c^
								0	363	0	378	Mortality rate^c^
Kara Polat et al. (2020) (24)	multicenter, prospective, cross-sectional	Istanbul	3/11/20-6/28/20	adults	Secukinumab and Ixekizumab	Topical therapies, Phototherapy, Acitretin, Methotrexate and Cyclosporine	suspected and confirmed cases	5	133	14	927	Infection rate^e^
								2	133	7	927	Hospitalization rate
								0	133	0	927	Mortality rate^c^
Kartal et al. (2021) (25)	multicenter, prospective, cross-sectional	Turkey	3/11/20-7/11/20	adults	Secukinumab and Ixekizumab	Methotrexate and Cyclosporine	confirmed cases	0	436	2	621	Infection rate^c^
								0	436	0	621	Hospitalization rate^c^
								0	436	0	621	Mortality rate^c^
Kridin et al. (2021) (26)	multicenter, retrospective, cohort	Israel	2/27/20-10/02/20	NA	Secukinumab and Ixekizumab	Methotrexate	confirmed cases	13	680	42	2153	Infection rate
								1	680	11	2153	Hospitalization rate
								1	680	1	2153	Mortality rate
Queiro et al. (2020) (27)	single-center, retrospective, case series	Spain	NA	NA	Secukinumab	Apremilast	confirmed cases	2	209	3	303	Infection rate
								1	209	2	303	Hospitalization rate
								0	209	0	303	Mortality rate^c^
Vispi et al. (2020) (28)	multicenter, retrospective, experience	Italy	3/01/20-4/30/20	adults	Secukinumab, Ixekizumab and Brodalumab	Apremilast	confirmed cases	0	52	0	22	Infection rate^c^
								0	52	0	22	Hospitalization rate^c^
								0	52	0	22	Mortality rate^c^

COVID-19, coronavirus disease 2019; IL-17, interleukin-17; NA, not available.

^a^Exposure represents the patients with psoriasis treated with IL-17 inhibitors.

^b^Control represents the patients with psoriasis treated with non-biologics.

^c^The studies used the fixed 0.5 correction value.

^d^Data provided by Dr. Fougerousse Anne-Claire. In the exposure group, there were 14 COVID-19 cases, including 2 confirmed cases and 12 suspected cases. And in the control group, there were 23 COVID-19 cases, including 3 confirmed cases (1 hospitalized with COVID-19) and 20 suspected cases.

^e^In the exposure group, there were 5 COVID-19 cases, including 3 confirmed cases, 1 suspected cases and 1 sample not gone. And in the control group, there were 14 COVID-19 cases, including 11 confirmed cases (5 hospitalized with COVID-19) and 3 suspected cases (2 hospitalized with COVID-19).

### Risk of bias assessment

**Figure 2:**
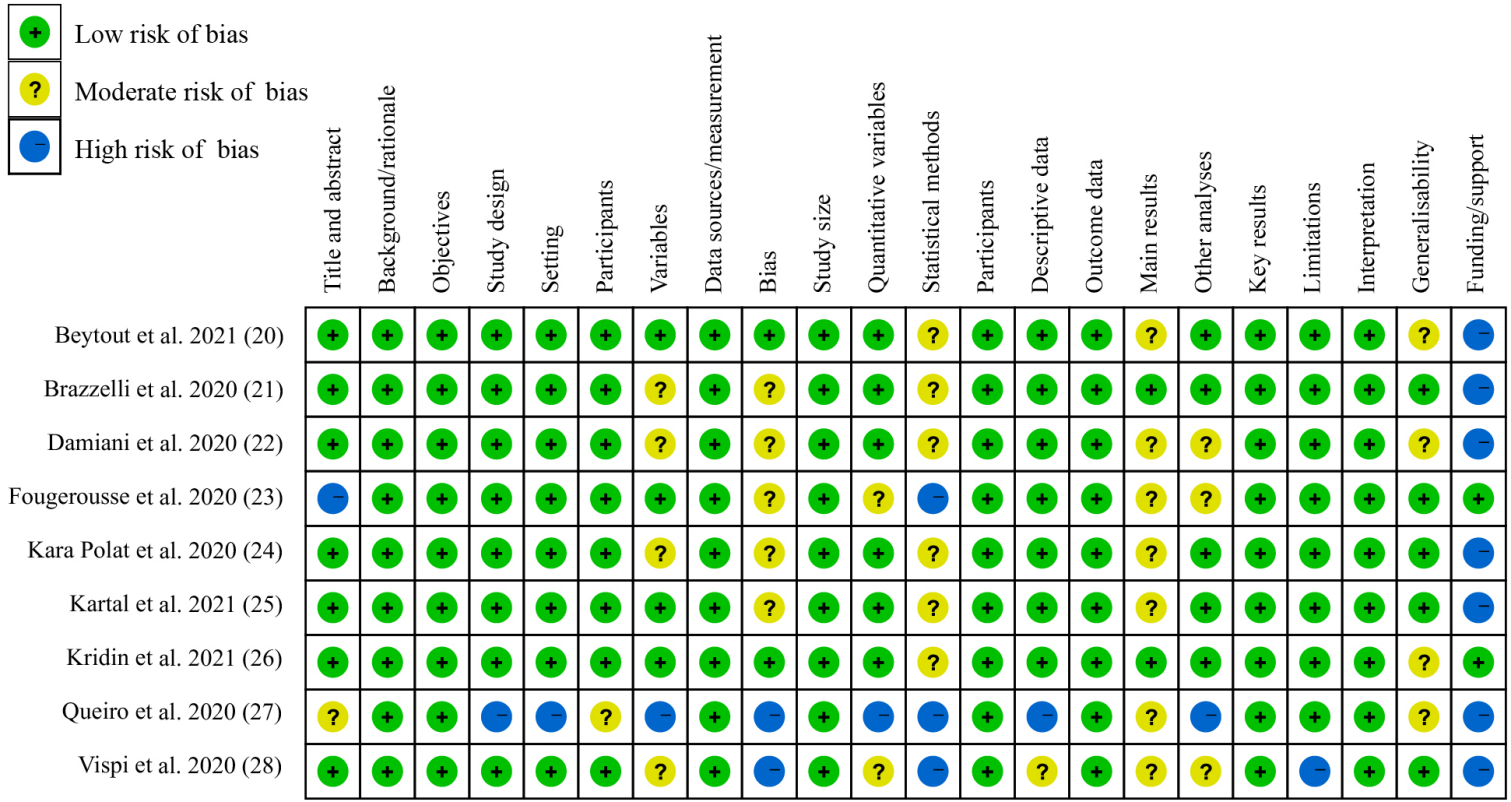
Risk of bias assessment.

All studies were observational, including three single-center studies and six multicenter studies. The quality of each study was assessed, and showed an overall low risk of bias rating, except for the study by Queiro et al. (27) (**Figure 2**). Because Queiro et al. conducted a case series study, it exhibited an overall moderate risk of bias rating.

### Effect of IL-17 inhibitors on the risk of SARS-CoV-2 infection

**Figure 3:**
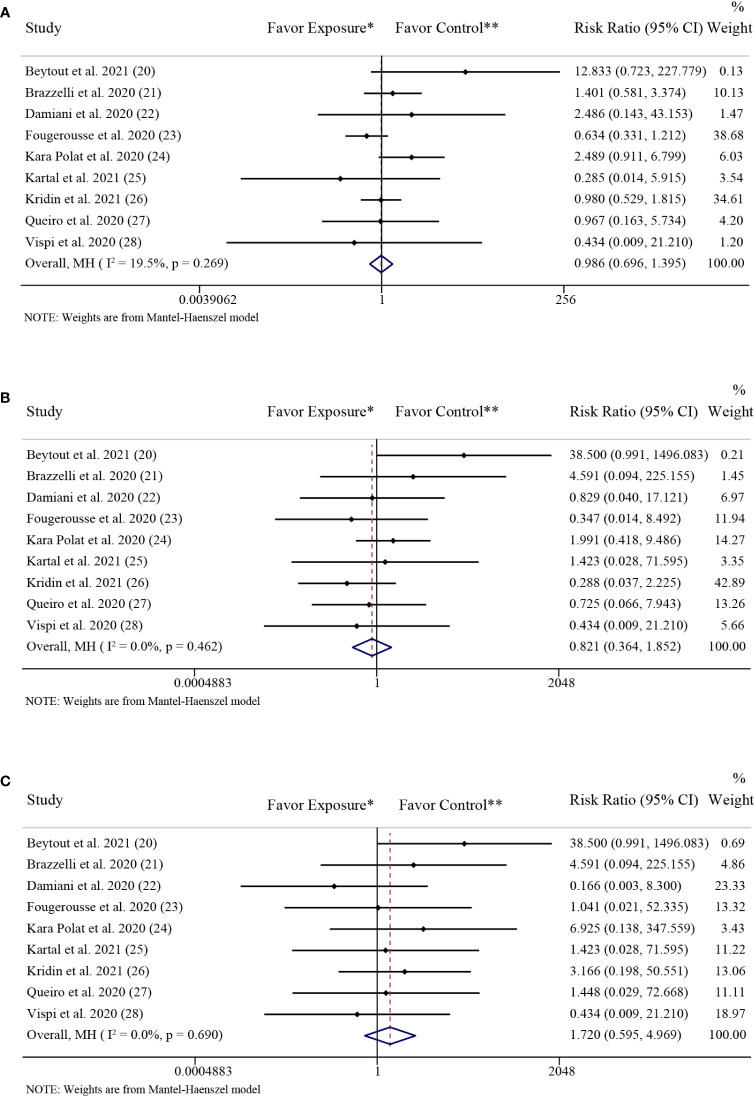
Forest plots of risk ratios and 95% confidence intervals. **(A)** Forest plot of risk ratios and 95% CIs of the SARS-CoV-2 infection rate; **(B)** Forest plot of risk ratios and 95% CIs of the COVID-19 hospitalization rate; **(C)** Forest plot of risk ratios and 95% CIs of the COVID-19 mortality rate. *CI*, confidence interval; *COVID-19*, coronavirus disease 2019; *MH*, Mantel-Haenszel method. ^*^Exposure represents patients with psoriasis treated with IL-17 inhibitors. ^**^Control represents patients with psoriasis treated with non-biologics.

**Figure 3** showed the forest plots of including studies investigating the differences in the rate of COVID-19 infection, hospitalization and mortality between the patients with psoriasis receiving IL-17 inhibitors (secukinumab, ixekizumab, or brodalumab) and those receiving non-biologics (topical therapies, phototherapy, acitretin, methotrexate, cyclosporine, apremilast and dimetil-fumarate). The pooled effects showed no significant difference in the rate of SARS-CoV-2 infection (RR: 0.99; 95% CI: 0.70-1.40; *P* = 0.94) (**Figure 3A**) between the two groups. The low level of heterogeneity was found in the studies investigating the rate of SARS-CoV-2 infection (*I* ^2^ = 19.5%; *P* = 0.27).

**Figure 4:**
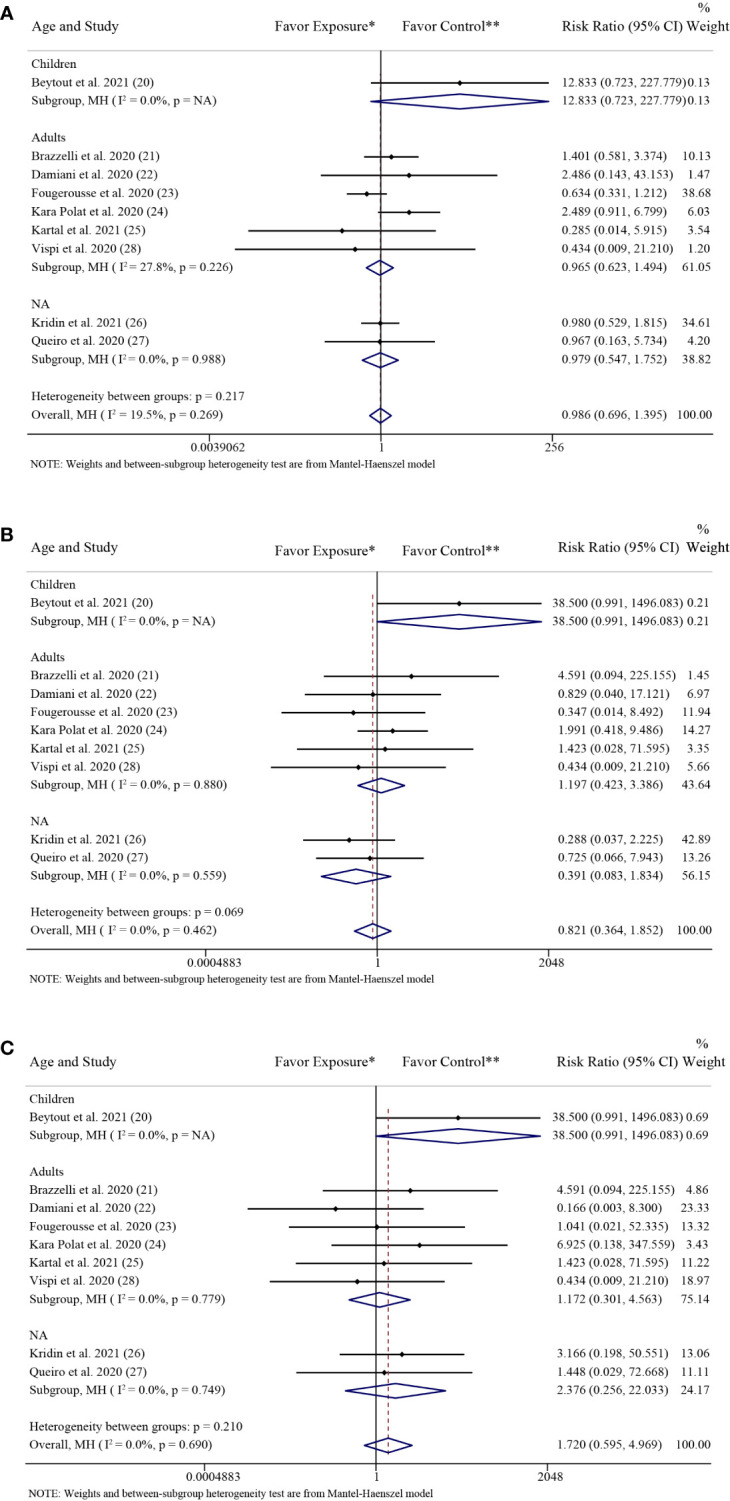
Subgroup analysis grouped by age. **(A)** Forest plot of risk ratios and 95% CIs of the SARS-CoV-2 infection rate; **(B)** Forest plot of risk ratios and 95% CIs of the COVID-19 hospitalization rate; **(C)** Forest plot of risk ratios and 95% CIs of the COVID-19 mortality rate. *CI*, confidence interval; *COVID-19*, coronavirus disease 2019; *MH*, Mantel-Haenszel method; *NA*, not available. ^*^Exposure represents patients with psoriasis treated with IL-17 inhibitors. ^**^Control represents patients with psoriasis treated with non-biologics.

The results of the subgroup analysis grouped by age were consistent with the above overall results. In the adults group, there was no significant difference in the rate of SARS-CoV-2 infection (RR: 0.97; 95% CI: 0.62-1.49; *P* = 0.87) between the patients with psoriasis receiving IL-17 inhibitors and those receiving non-biologics. There were also no significant differences in the rate of SARS-CoV-2 infection between the patients with psoriasis receiving IL-17 inhibitors and those receiving non-biologics in the children group (RR: 12.83; 95% CI: 0.72-227.78; *P* = 0.08) and the NA group (RR: 0.98; 95% CI: 0.55-1.75; *P* = 0.94), respectively (**Figure 4A**).

**Figure 5:**
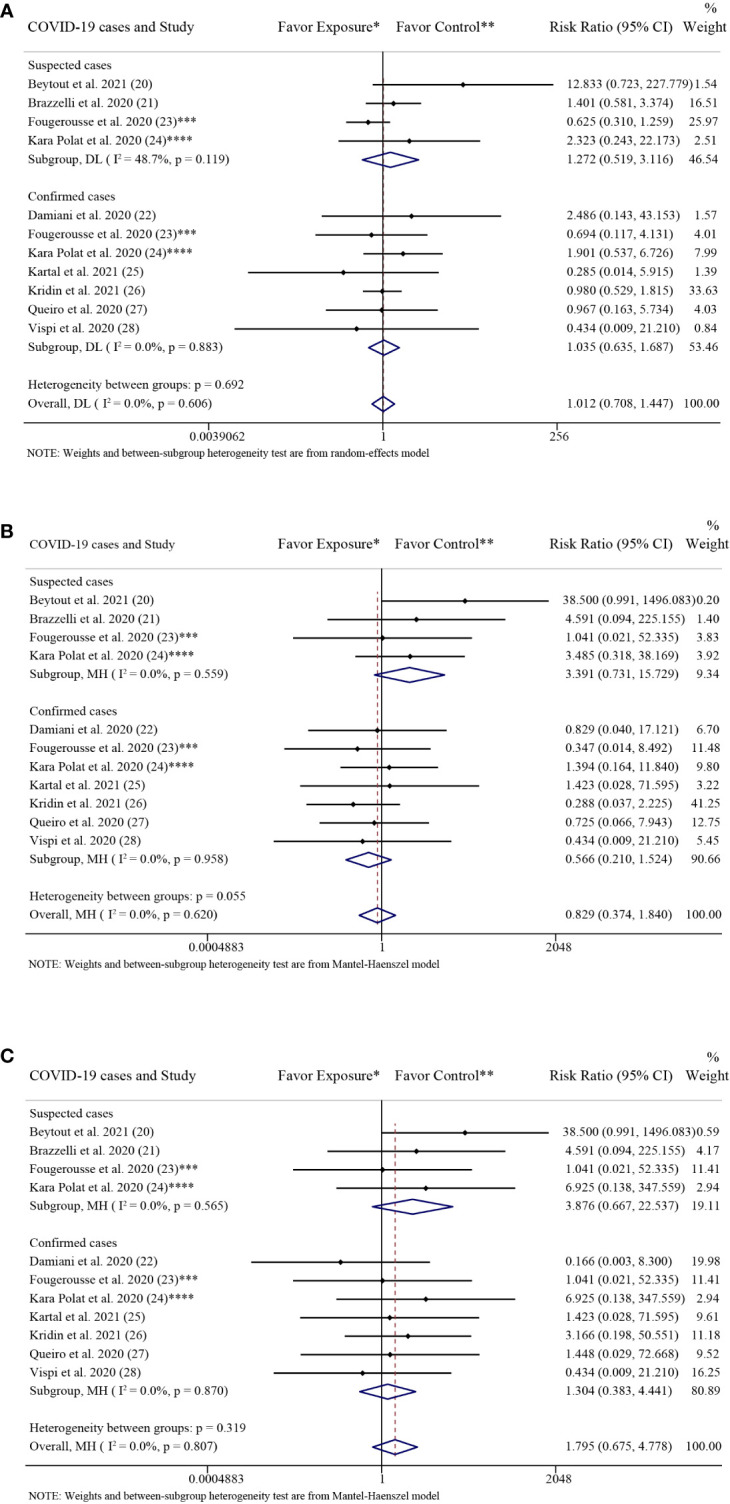
Subgroup analysis grouped by COVID-19 cases. **(A)** Forest plot of risk ratios and 95% CIs of the SARS-CoV-2 infection rate; **(B)** Forest plot of risk ratios and 95% CIs of the COVID-19 hospitalization rate; **(C)** Forest plot of risk ratios and 95% CIs of the COVID-19 mortality rate. *CI*, confidence interval; *COVID-19*, coronavirus disease 2019; *DL*, DerSimonian-Laird estimate of tau^2^; *NA*, not available. ^*^Exposure represents patients with psoriasis treated with IL-17 inhibitors. ^**^Control represents patients with psoriasis treated with non-biologics. ^***^In the exposure group, there were 14 COVID-19 cases, including 2 confirmed cases and 12 suspected cases. And in the control group, there were 23 COVID-19 cases, including 3 confirmed cases (1 hospitalized with COVID-19) and 20 suspected cases. ^****^In the exposure group, there were 5 COVID-19 cases, including 3 confirmed cases, 1 suspected cases and 1 sample not gone. And in the control group, there were 14 COVID-19 cases, including 11 confirmed cases (5 hospitalized with COVID-19) and 3 suspected cases (2 hospitalized with COVID-19).

The results of the subgroup analysis grouped by COVID-19 cases were consistent with the above overall results. There were no significant differences in the rate of SARS-CoV-2 infection between the patients with psoriasis receiving IL-17 inhibitors and those receiving non-biologics in the confirmed cases group (RR: 1.04; 95% CI: 0.64-1.69; *P* = 0.89) and in the suspected cases group (RR: 1.27; 95% CI: 0.52-3.12; *P* = 0.60), respectively (**Figure 5A**).

In the separate comparative analyses, the results showed no significant differences in the rate of SARS-CoV-2 infection in psoriasis patients receiving topical therapies, phototherapy, apremilast, dimetil-fumarate, acitretin, methotrexate or cyclosporine alone compared to those receiving IL-17 inhibitors (**Figure S1**).

Sensitivity analysis showed the stability of the results regarding the risk of SARS-CoV-2 infection (**Table 2**) (**Figure S4A**). There was no visual asymmetry in the funnel plot (**Figure S5A**). Neither Egger’s test (*P* = 0.46) (**Figure S6A**) nor Begg’s test (*P* = 1.00) (**Figure S7A**) revealed publication bias (**Table 2**).

**Table 2:** Summary of Analysis Results.

	Infection rate of SARS-CoV-2				Hospitalization rate of COVID-19				Mortality rate of COVID-19			
**Sensitivity analysis**	Study omitted	Estimate (RR)	95% CI		Study omitted	Estimate (RR)	95% CI		Study omitted	Estimate (RR)	95% CI	
	Beytout et al. (2021) (20)	0.970	0.683	1.377	Beytout et al. (2021) (20)	0.743	0.316	1.748	Beytout et al. (2021) (20)	1.465	0.464	4.627
	Brazzelli et al. (2020) (21)	0.939	0.644	1.369	Brazzelli et al. (2020) (21)	0.765	0.329	1.779	Brazzelli et al. (2020) (21)	1.573	0.516	4.799
	Damiani et al. (2020) (22)	0.963	0.679	1.367	Damiani et al. (2020) (22)	0.820	0.353	1.909	Damiani et al. (2020) (22)	2.193	0.673	7.143
	Fougerousse et al. (2020) (23)	1.208	0.796	1.833	Fougerousse et al. (2020) (23)	0.885	0.379	2.065	Fougerousse et al. (2020) (23)	1.824	0.608	5.469
	Kara Polat et al. (2020) (24)	0.889	0.612	1.292	Kara Polat et al. (2020) (24)	0.626	0.234	1.673	Kara Polat et al. (2020) (24)	1.535	0.498	4.726
	Kartal et al. (2021) (25)	1.011	0.712	1.436	Kartal et al. (2021) (25)	0.800	0.348	1.841	Kartal et al. (2021) (25)	1.757	0.588	5.253
	Kridin et al. (2021) (26)	0.989	0.650	1.504	Kridin et al. (2021) (26)	1.221	0.497	3.000	Kridin et al. (2021) (26)	1.503	0.472	4.781
	Queiro et al. (2020) (27)	0.986	0.692	1.406	Queiro et al. (2020) (27)	0.836	0.352	1.984	Queiro et al. (2020) (27)	1.754	0.587	5.243
	Vispi et al. (2020) (28)	0.992	0.700	1.407	Vispi et al. (2020) (28)	0.844	0.367	1.943	Vispi et al. (2020) (28)	2.021	0.652	6.267
	Combined	0.986	0.696	1.395	Combined	0.821	0.364	1.852	Combined	1.720	0.595	4.969
**Publication bias** ^a^	*p* value (Begg’s test)		*p* value (Egger’s test)		*p* value (Begg’s test)		*p* value (Egger’s test)		*p* value (Begg’s test)		*p* value (Egger’s test)	
	1.00		0.46		0.60		0.69		0.47		0.53	
**NNT** ^b^	NNTB (Total)	No. of avoided events per 1000 (CI)			NNTB (Total)	No. of avoided events per 1000 (CI)			NNTH (Total)	No. of excess events per 1000 (CI)		
	3247	1 (-9, 7)			1118	1 (-5, 4)			1389	1 (-1, 4)		

CI, confidence interval; COVID-19, coronavirus disease 2019; NA, not available; NNT, number needed to treat; NNTB, number needed to treat to benefit; NNTH, number needed to treat to harm; RR, risk ratio; SARS-CoV-2, severe acute respiratory syndrome coronavirus 2.

^a^ Effect size: Ln (risk ratio).

^b^ Values rounded up to the nearest integer.

The pooled NNT showed no significant difference in the clinical value of preventing SARS-CoV-2 infection in the psoriasis patients receiving IL-17 inhibitors and those receiving non-biologics (**Table 2**). The result of NNT was consistent with the result of the RR.

### Effect of IL-17 inhibitors on the risk of COVID-19 hospitalization

The pooled effects showed no significant difference in the rate of COVID-19 hospitalization (RR: 0.82; 95% CI: 0.36-1.85; *P* = 0.64) (**Figure 3B**) between the patients with psoriasis receiving IL-17 inhibitors and those receiving non-biologics. The low level of heterogeneity was found in the studies investigating the rate of COVID-19 hospitalization (*I* ^2^ = 0.0%; *P* = 0.46).

The results of the subgroup analysis grouped by age were consistent with the above overall results. In the adults group, there was no significant difference in the rate of COVID-19 hospitalization (RR: 1.20; 95% CI: 0.42-3.39; *P* = 0.74) between the patients with psoriasis receiving IL-17 inhibitors and those receiving non-biologics. There were also no significant differences in the rate of COVID-19 hospitalization between the patients with psoriasis receiving IL-17 inhibitors and those receiving non-biologics in the children group (RR: 38.50; 95% CI: 0.99-1496.08; *P* = 0.05) and the NA group (RR: 0.39; 95% CI: 0.08-1.83; *P* = 0.23), respectively (**Figure 4B**).

The results of the subgroup analysis grouped by COVID-19 cases were consistent with the above overall results. There were no significant differences in the rates of COVID-19 hospitalization between the patients with psoriasis receiving IL-17 inhibitors and those receiving non-biologics in the confirmed cases group (RR: 0.57; 95% CI: 0.21-1.52; *P* = 0.26) and in the suspected cases group (RR: 3.39; 95% CI: 0.73-15.73; *P* = 0.12), respectively (**Figure 5B**).

In the separate comparative analyses, the results also showed no significant differences in the rate of COVID-19 hospitalization in psoriasis patients receiving any of the specific non-biologics alone compared to those receiving IL-17 inhibitors (**Figure S2**).

Sensitivity analysis showed the stability of the results regarding the risk of COVID-19 hospitalization (**Table 2**) (**Figure S4B**). There was no visual asymmetry in the funnel plot (**Figure S5B**). Neither Egger’s test (*P* = 0.69) (**Figure S6B**) nor Begg’s test (*P* = 0.60) (**Figure S7B**) revealed publication bias (**Table 2**). The pooled NNT showed no significant difference in the clinical value of alleviating the course of COVID-19 in psoriasis patients receiving IL-17 inhibitors and those receiving non-biologics (**Table 2**). The result of NNT was consistent with the result of the RR.

### Effect of IL-17 inhibitors on the risk of COVID-19 mortality

The pooled effects showed no significant difference in the rate of COVID-19 mortality (RR: 1.72; 95% CI: 0.60-4.97; *P* = 0.32) (**Figure 3C**) between the patients with psoriasis receiving IL-17 inhibitors and those receiving non-biologics. The low level of heterogeneity was found in the studies investigating the rate of COVID-19 mortality (*I* ^2^ = 0.0%; *P* = 0.69).

The results of the subgroup analysis grouped by age were consistent with the above overall results. In the adults group, there was no significant difference in the rate of COVID-19 mortality (RR: 1.17; 95% CI: 0.30-4.56; *P* = 0.82) between the patients with psoriasis receiving IL-17 inhibitors and those receiving non-biologics. There were also no significant differences in the rate of COVID-19 mortality between the patients with psoriasis receiving IL-17 inhibitors and those receiving non-biologics in the children group (RR: 38.50; 95% CI: 0.99-1496.08; *P* = 0.05) and the NA group (RR: 2.38; 95% CI: 0.26-22.03; *P* = 0.45), respectively (**Figure 4C**).

The results of the subgroup analysis grouped by COVID-19 cases were consistent with the above overall results. There were no significant differences in the rate of COVID-19 mortality between the patients with psoriasis receiving IL-17 inhibitors and those receiving non-biologics in the confirmed cases group (RR: 1.30; 95% CI: 0.38-4.44; *P* = 0.67) and in the suspected cases group (RR: 3.88; 95% CI: 0.67-22.54; *P* = 0.13), respectively (**Figure 5C**).

In the separate comparative analyses, the results also showed no significant difference in the rate of COVID-19 mortality in psoriasis patients receiving any of the specific non-biologics alone compared to those receiving IL-17 inhibitors (**Figure S3**).

Sensitivity analysis showed the stability of the results regarding the risk of COVID-19 mortality (**Table 2**) (**Figure S4C**). There was no visual asymmetry in the funnel plot (**Figure S5C**). Neither Egger’s test (*P* = 0.53) (**Figure S6C**) nor Begg’s test (*P* = 0.47) (**Figure S7C**) revealed publication bias (**Table 2**). The pooled NNT showed no significant difference in the clinical value of treating COVID-19 disease in psoriasis patients receiving IL-17 inhibitors and those receiving non-biologics (**Table 2**). The result of NNT was consistent with the result of the RR.

## Discussion

This meta-analysis, including both single-center and multicenter observational studies, compared the risk of COVID-19 infection, hospitalization, and mortality in patients with psoriasis receiving IL-17 inhibitors and those receiving non-biologics. We found no significant differences in the rates of COVID-19 infection, hospitalization and mortality between the two groups. These findings indicated that using IL-17 inhibitors in psoriasis patients did not increase the infection risk of SARS-CoV-2 or worsen the course of COVID-19 compared with using non-biologics.

IL-17 is selectively produced by a specific subset of T helper cells–Th17, and is identified as a silent amplifier of the immunity process (29). IL-17 plays an important role in some inflammatory-based chronic diseases (30), and is involved in the process of cell activation, growth and proliferation (31, 32). IL-17 could induce the production of pro-inflammatory mediators such as IL-6 and tumor necrosis factor (TNF)-α (33). Studies showed that the levels of Th17 cells and IL-17 in the peripheral blood of patients infected with SARS-CoV-2 were elevated (2, 6–8). Also, elevated plasma levels of these pro-inflammatory mediators have also been associated with COVID-19-related pulmonary inflammation (2, 34). Study had shown that 81% of COVID-19 fatal cases were diagnosed with acute respiratory distress syndrome (ARDS) (35), which indicated ARDS was an important cause of COVID-19 death. Meanwhile, a retrospective analysis of IL-17 gene polymorphisms showed an increased 30-day survival in ARDS patients with the A-allele of rs2275913 SNP (resulting in attenuated IL-17 production) (36). Additionally, a study revealed that in COVID-19 neutrophil/T cell cocultures, neutrophils could cause a strong polarity shift to Th17, and was accompanied by a decrease of interferon (IFN)-γ-producing Th1 cells (37), which confirmed the association between Th17 and COVID-19. Moreover, a Th17 type-dominant immunophenotype could drive more severe viral myocarditis, which might further increase the risk of COVID-19 mortality (38). Together, these studies revealed the potential relation between elevated IL-17 level and the severity and progression of COVID-19, and it might be a possible effective measure for prevention of SARS-CoV-2 infection or treatment of COVID-19 to use existing IL-17 inhibitors. All currently approved IL-17 inhibitors (ixekizumab and secukinumab, both anti-IL-17A monoclonal antibody; brodalumab, anti-IL-17RA monoclonal antibody) can target IL-17 directly or indirectly. However, IL-17 inhibitors have been reported to increase the risk of respiratory infections. A study indicated that IL-17 inhibitors increased the overall risk of infections up to 11%, with much of the upper respiratory infections could be attributed to secukinumab, not ixekizumab or brodalumab (17). So there was a concern that the psoriasis patients treated with IL-17 inhibitors might be more susceptible to the viral infection or develop a more severe disease. To date, there is still a lack of sufficient evidence to elucidate the effect of IL-17 inhibitors on COVID-19 susceptibility and severity.

In this meta-analysis, we assessed the effect of IL-17 inhibitors on the risk of COVID-19 infection, hospitalization, and mortality in psoriasis patients by comparing them to psoriasis patients receiving non-biologics. These non-biologics included topical therapies, phototherapy, apremilast, dimetil-fumarate, acitretin, methotrexate and cyclosporine. Among them, methotrexate, cyclosporine and apremilast are of great attention because they are immunosuppressive agents, especially methotrexate. Comparatively, topical therapies, phototherapy, acitretin and dimetil-fumarate do not seem to cause undue concern during the COVID-19 pandemic.

Methotrexate is an inhibitor of dihydrofolate reductase that suppresses inflammation and immune infections. Previous studies have shown that methotrexate can increase infection (39), but its effect on susceptibility/severity of COVID-19 is unclear. Kridin et al. found higher rates of COVID-19 hospitalization in psoriasis patients exposed to methotrexate than to TNF inhibitors, but similar rates of SARS-CoV-2 infection (34), and that methotrexate intake independently predicted COVID-19 related hospitalizations (40). Also, Izadi et al. observed a higher risk of hospitalization or death associated with COVID-19 among patients with immune-mediated inflammatory diseases (IMIDs) treated with methotrexate compared with TNF inhibitors, but notably, patients treated with methotrexate in combination with TNF inhibitors had similar rates of hospitalization or death to those treated with TNF inhibitors alone, and importantly, methotrexate determined COVID-19 outcomes in the combination regimen (41). This suggests that in COVID-19, methotrexate acts in the same direction as TNF inhibitors. More importantly, data from a global electronic database (42) showed no statistically significant difference in hospitalization rates in COVID-19 patients exposed to methotrexate compared to those not exposed to methotrexate. Additionally, Ganjei et al. (43) found that patients with aplastic anemia taking low dose methotrexate had significantly less severe symptoms of COVID-19 than their families with normal immune systems. This demonstrates a potential protective effect of methotrexate on the progression of COVID-19. Some researchers have even speculated that methotrexate may inhibit the expression of TNF-α, IL-6 and other pro-inflammatory cytokines released with SARS-CoV-2, thereby reducing the cytokine storm associated with COVID-19 (44, 45). Taken together, these evidences suggest that methotrexate most likely does not increase the susceptibility or severity of COVID-19 and may even protect patients from severe COVID-19. Our results showed similar risks of COVID-19 infection, hospitalization and mortality in psoriasis patients under IL-17 inhibitors compared with those under methotrexate, which indirectly demonstrated the potential protective effect of IL-17 inhibitors on COVID-19 infection and progression in patients with psoriasis.

Cyclosporine is a calcineurin inhibitor and premilast is a phosphodiesterase-4 inhibitor, both of which modulate immune response. Studies showed that non-cytotoxic concentrations of cyclosporine strongly inhibited replication of several coronaviruses *in vitro*, including SARS-CoV, MERS-CoV and HCoV-229E (46–48), and that the psoriasis patients taking apremilast had a significantly lower rate of severe viral infection (49). Interestingly, cyclosporine and apremilast probably reduce the risk of cytokine storm associated with SARS-CoV infection by downregulating the expression of pro-inflammatory cytokines (50, 51). More importantly, several studies (27, 52, 53) have observed a favorable safety profile of apremilast on COVID-19 susceptibility/severity in patients with psoriasis. Dimetil-fumarate is a small molecule with potent, broad-spectrum antibacterial properties, and acitretin has anti-inflammatory effects and inhibits cell differentiation. Studies indicated that acitretin did not increase risk of viral or respiratory infections in psoriasis patients (49), and the rates of COVID-19 infection and hospitalization in psoriasis patients exposed to acitretin were similar to that of exposure to TNF inhibitors (34). Topical therapies and phototherapy have shown a good safety profile, and remain an important option for psoriasis patients during the COVID-19 pandemic. In conclusion, these studies strongly suggest that these non-biologics do not increase susceptibility or severity of COVID-19, which also confirm the rationality and scientific validity of using them as the control group in this meta-analysis to assess the effect of IL-17 inhibitors on the risk of COVID-19 infection, hospitalization and mortality.

Furthermore, in two studies including 119 and 346 patients with psoriasis using IL-17 inhibitors, respectively, none was infected with SARS-CoV-2 (54, 55), which an evidence that the use of IL-17 inhibitors in psoriasis could not increase the risk of SARS-CoV-2 infection. These studies were consistent with the results of our meta-analysis that the use of IL-17 inhibitors in psoriasis patients did not more prone to SARS-CoV-2. Meanwhile, a study showed that the use of biologics (including IL-17 inhibitors) in psoriasis patients did not increase susceptibility to contracting COVID-19, severe disease progression, or increased hospitalization and mortality rates (56). Moreover, psoriasis patients receiving biologics (including IL-17 inhibitors) were reported to have a lower hospitalization rate than those receiving non-biologics (acitretin, apremilast, cyclosporine, etc.) (57). Although this was not shown in our meta-analysis, it undoubtedly reinforced the viewpoint that the use of IL-17 inhibitors in psoriasis patients did not exacerbate COVID-19 conditions. At present, clinical data on IL-17 inhibitors for the treatment of COVID-19 are still scarce, with only a few case reports showing the efficacy and safety of IL-17 inhibitors in patients with COVID-19. Di Lernia et al. (58) and Balestri et al. (59) have reported favorable outcomes with secukinumab/ixekizumab in patients with COVID-19, respectively. Mugheddu et al. (60) found that secukinumab could even be safely treat severe COVID-19 patients and shorten their disease course. Additionally, other reports (61–63) also showed the similar results. Surprisingly, Carugno et al. (64) demonstrated an experience with a COVID-19 psoriatic patient treated with secukinumab who developed a late onset rash. At the onset of the rash, the patient had a nasopharyngeal positive swab, but the RT-PCR search for viruses in skin was negative, which further supported the potential therapeutic use of IL-17 inhibitors in COVID-19. Overall, these previous studies showed the safety of using IL-17 inhibitors in patients with psoriasis in the setting of COVID-19 pandemic, and further supported the positive effect of IL-17 inhibitors on alleviating the course of COVID-19. And the results of our meta-analysis largely coincided with it.

Large epidemiological studies showed that psoriasis was one of the most common dermatological diseases in patients with COVID-19 (65), and that patients with psoriasis were more susceptible to the SARS-CoV-2 and had a higher risk of dying from COVID-19 (66, 67). However, it was also reported that psoriasis alone (without its comorbidities) was not likely to be a risk factor for the severity of SARA-COV-2 infection (68). Currently, the relationship between psoriasis and COVID-19 is still not entirely clear. National Psoriasis Foundation COVID-19 Task Force (NPF COVID-19 TF) reached a high level of consensus that the likelihood of poor outcomes from COVID-19 is driven by comorbidities such as chronic heart, lung or kidney disease and metabolic disorders such as diabetes and obesity. And psoriasis patients are more prone to these comorbidities, particularly in those with more severe disease (69). Interestingly, psoriasis shares a similar immune-inflammatory mechanism with cardiovascular diseases, both involving activation of Th1 and Th17 cells and decreased T-regulatory cell function (70, 71). Studies have shown that the application of IL-17 inhibitors can improve psoriasis and cardiovascular disease (72, 73). Therefore, it further supported that IL-17 inhibitors played a significant positive role in the prevention of SARS-CoV-2 infection and COVID-19 worsening. It is well known that psoriasis is a complex disease, and it’s considered quite harmful to hastily interrupt treatment (74). On one hand, it will lead to higher pro-inflammatory states which can potentially worsen the cytokine storm and immune responses to SARS-CoV-2. On the other hand, discontinuation of biological therapies may cause increased medical costs due to the inevitable recurrence of disease, for the efficacy may be reduced if the patient uses the same biological therapy again after discontinuation (75, 76). Our study supports the continued use of IL-17 inhibitors in patients with psoriasis during the COVID-19 pandemic, which avoids potential exacerbations of psoriasis due to treatment interruption.

This research has several limitations. Firstly, due to the novelty of the research topic and the lack of randomized controlled trials data, we eventually included observational studies such as case series, cross-sectional studies, and cohort studies, which may result in a downgrade of research evidence. Secondly, individual characteristics can affect the risk of SARS-CoV-2 infection and course of COVID-19, such as gender, comorbidities, and compliance with epidemic prevention measures. And due to the lack of these elements in the included studies, our study did not address them. These limitations may be sources for heterogeneity.

Nonetheless, there are some strengths in our meta-analysis. Firstly, our study is based on observational studies that investigate psoriasis patients in their natural state, which provides certain guidance to dermatologists in their clinical management. Secondly, we included thousands of individuals, which increased the credibility of this meta-analysis. Thirdly, we performed a more comprehensive analysis, including overall analysis and subgroup analyses grouped by age and COVID-19 cases, respectively, and further confirmed that the use of IL-17 inhibitors in psoriasis did not increase the risk of SARS-CoV-2 infection or worsen the course of COVID-19 compared with the use of non-biologics.

## Conclusion

Our meta-analysis study with a large sample size of 2,437 psoriasis patients using IL-17 inhibitors and 4,669 psoriasis patients using non-biologics strongly support that the use of IL-17 inhibitors in patients with psoriasis does not increase the risk of SARS-CoV-2 infection or worsen the course of COVID-19. This review provides evidence to support treatment decision making for psoriasis patients receiving IL-17 inhibitors in the COVID-19 pandemic. Well-controlled clinical trials are warranted to demonstrate the efficacy and safety of IL-17 inhibitors in the treatment of COVID-19 in the psoriasis population.

## Data availability statement

The original contributions presented in the study are included in the article/**Supplementary Material**. Further inquiries can be directed to the corresponding authors.

## Author contributions

ML had full access to all of the data in the study and took responsibility for the integrity of the data and the accuracy of the data analysis. Concept and design: ML, GZ, NW. Acquisition, analysis, or interpretation of data: GZ, NW, LL, SC, BW, ML. Drafting of the manuscript: ML, HW, XH, ZX, YZ. Critical revision of the manuscript for important intellectual content: All authors. Statistical analysis: ML, SC, LL. Supervision: GZ, NW. All authors contributed to the article and approved the submitted version.

## Acknowledgments

We sincerely thank Dr. Fougerousse Anne-Claire for her data support.

## Conflict of interest

The authors declare that the research was conducted in the absence of any commercial or financial relationships that could be construed as a potential conflict of interest.

## Publisher’s note

All claims expressed in this article are solely those of the authors and do not necessarily represent those of their affiliated organizations, or those of the publisher, the editors and the reviewers. Any product that may be evaluated in this article, or claim that may be made by its manufacturer, is not guaranteed or endorsed by the publisher.
