# Supplementary material for: Risk of COVID-19 infection, hospitalization and mortality in psoriasis patients treated with interleukin-17 inhibitors: A systematic review and meta-analysis
Source: Front Immunol. 2022 Oct 21;13:1046352. doi: 10.3389/fimmu.2022.1046352 (PMC9648142; doi:10.3389/fimmu.2022.1046352)
Supplement: Supplementary file 1 [file DataSheet_1.docx]

Supplementary Material

**Supplementary Table 1.** Retrieval Strategies Used to Filter the Literature in Each Database.

| **Database** | **Retrieval Strategies** | **Result** |
| --- | --- | --- |
| 1.Embase | #1: 'severe acute respiratory syndrome coronavirus 2'/exp  #2: 'coronavirus disease 2019'/exp  #3: 'coronavirus disease 2019':ti,ab,kw OR 'severe acute respiratory syndrome coronavirus 2':ti,ab,kw  #4: 'psoriasis'/exp  #5: psoriasis:ti,ab,kw OR psoriases:ti,ab,kw  #6: 'interleukin 17'/exp  #7: 'secukinumab'/exp  #8: 'ixekizumab'/exp  #9: 'brodalumab'/exp  #10: 'interleukin 17':ti,ab,kw OR secukinumab:ti,ab,kw OR ixekizumab:ti,ab,kw OR brodalumab:ti,ab,kw OR cosentyx:ti,ab,kw OR taltz:ti,ab,kw OR siliq:ti,ab,kw OR kyntheum:ti,ab,kw OR lumicef:ti,ab,kw  #11: #1 OR #2 OR #3  #12: #4 OR #5  #13: #6 OR #7 OR #8 OR #9 OR #10  #14: #11 AND #12 AND #13 | 261 |
| 2.PubMed | #1: "COVID-19"[MeSH Terms] OR "SARS-CoV-2"[MeSH Terms] OR "COVID-19"[Title/Abstract] OR "SARS-CoV-2"[Title/Abstract]  #2: "psoriasis"[MeSH Terms] OR "psoriasis"[Title/Abstract] OR "psoriases"[Title/Abstract]  #3: "Interleukin-17"[MeSH Terms] OR "il-17"[Title/Abstract] OR "Secukinumab"[Supplementary Concept] OR "Ixekizumab"[Supplementary Concept] OR "Brodalumab"[Supplementary Concept] OR "Cosentyx"[Title/Abstract] OR "Taltz"[Title/Abstract] OR "Siliq"[Title/Abstract] OR "Kyntheum"[Title/Abstract] OR "Lumicef"[Title/Abstract]  #4: #1 AND #2 AND #3 | 38 |
| 3.SCI-Web of Science | #1: TS=(COVID-19 OR SARS-CoV-2)  #2: TS=(psoriasis OR psoriases)  #3: TS=(interleukin 17 OR il-17 OR Secukinumab OR Cosentyx OR Ixekizumab OR Taltz OR Brodalumab OR Siliq OR Kyntheum OR Lumicef)  #4: #1 AND #2 AND #3 | 81 |
| 4.Scopus | #1: TITLE-ABS-KEY("COVID-19") OR TITLE-ABS-KEY("SARS-CoV-2")  #2: TITLE-ABS-KEY(psoriasis) OR TITLE-ABS-KEY(psoriases)  #3: TITLE-ABS-KEY("Interleukin-17") OR TITLE-ABS-KEY("il-17") OR TITLE-ABS-KEY(Secukinumab) OR TITLE-ABS-KEY(Ixekizumab) OR TITLE-ABS-KEY(Brodalumab) OR TITLE-ABS-KEY(Cosentyx) OR TITLE-ABS-KEY(Taltz) OR TITLE-ABS-KEY(Siliq) OR TITLE-ABS-KEY(Kyntheum) OR TITLE-ABS-KEY(Lumicef)  #4: #1 AND #2 AND #3 | 199 |
| 5.The Cochrane Library | #1: MeSH descriptor: [COVID-19] explode all trees  #2: MeSH descriptor: [SARS-CoV-2] explode all trees  #3: MeSH descriptor: [Psoriasis] explode all trees  #4: MeSH descriptor: [Interleukin-17] explode all trees  #5: (COVID-19 OR SARS-CoV-2):ti,ab,kw (Word variations have been searched)  #6: (psoriasis OR psoriases):ti,ab,kw (Word variations have been searched)  #7: (interleukin 17 OR il-17 OR Secukinumab OR Cosentyx OR Ixekizumab OR Taltz OR Brodalumab OR Siliq OR Kyntheum OR Lumicef):ti,ab,kw (Word variations have been searched)  #8: #1 OR #2 OR #5  #9: #3 OR #6  #10: #4 OR #7  #11: #8 AND #9 AND #10 | 5 |
| 6.CNKI | #1:（篇关摘：COVID-19 + SARS-CoV-2（模糊））OR（篇关摘：新型冠状病毒肺炎 + 新冠肺炎（模糊））  #2:（篇关摘：psoriasis（模糊））OR（篇关摘：银屑病 + 牛皮癣（模糊））  #3:（篇关摘：Secukinumab + Cosentyx + Ixekizumab + Taltz + Brodalumab + Siliq +Kyntheum+ Lumicef（模糊））OR（篇关摘：IL-17（模糊））OR（篇关摘：IL 17（模糊））OR（篇关摘：白介素-17（模糊））OR（篇关摘：白介素17（模糊））  #4 :#1 AND #2 AND #3 | 25 |


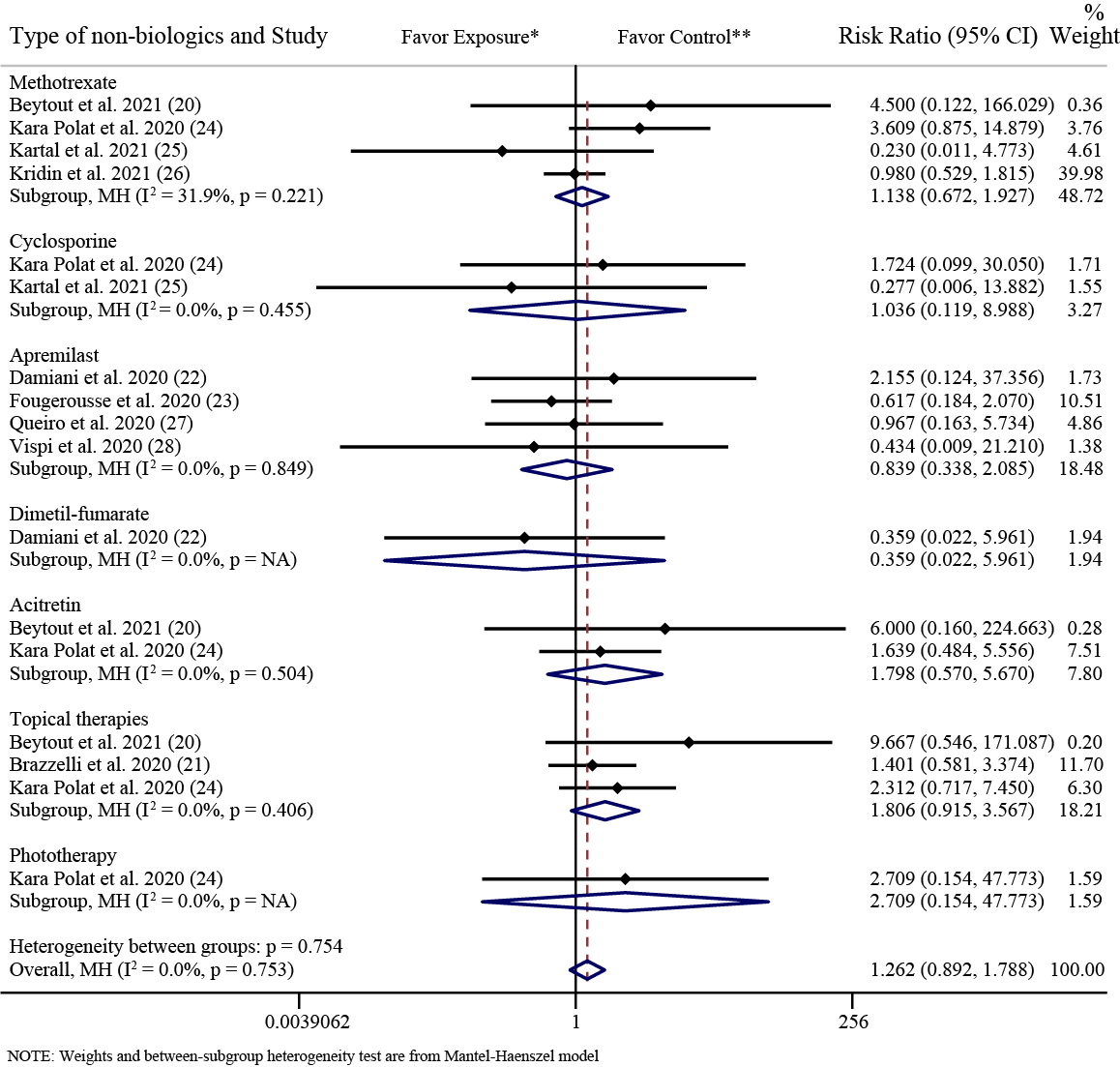


**Supplementary Figure 1**. Forest plot of risk ratios and 95% CIs of the SARS-CoV-2 infection rate in comparative analyses (grouped by the specific types of non-biologics). *CI*, confidence interval; *MH*, Mantel-Haenszel method; *NA*, not available; *SARS-CoV-2*, severe acute respiratory syndrome coronavirus 2. ^*^Exposure represents patients with psoriasis treated with IL-17 inhibitors. ^**^Control represents patients with psoriasis treated with non-biologics.


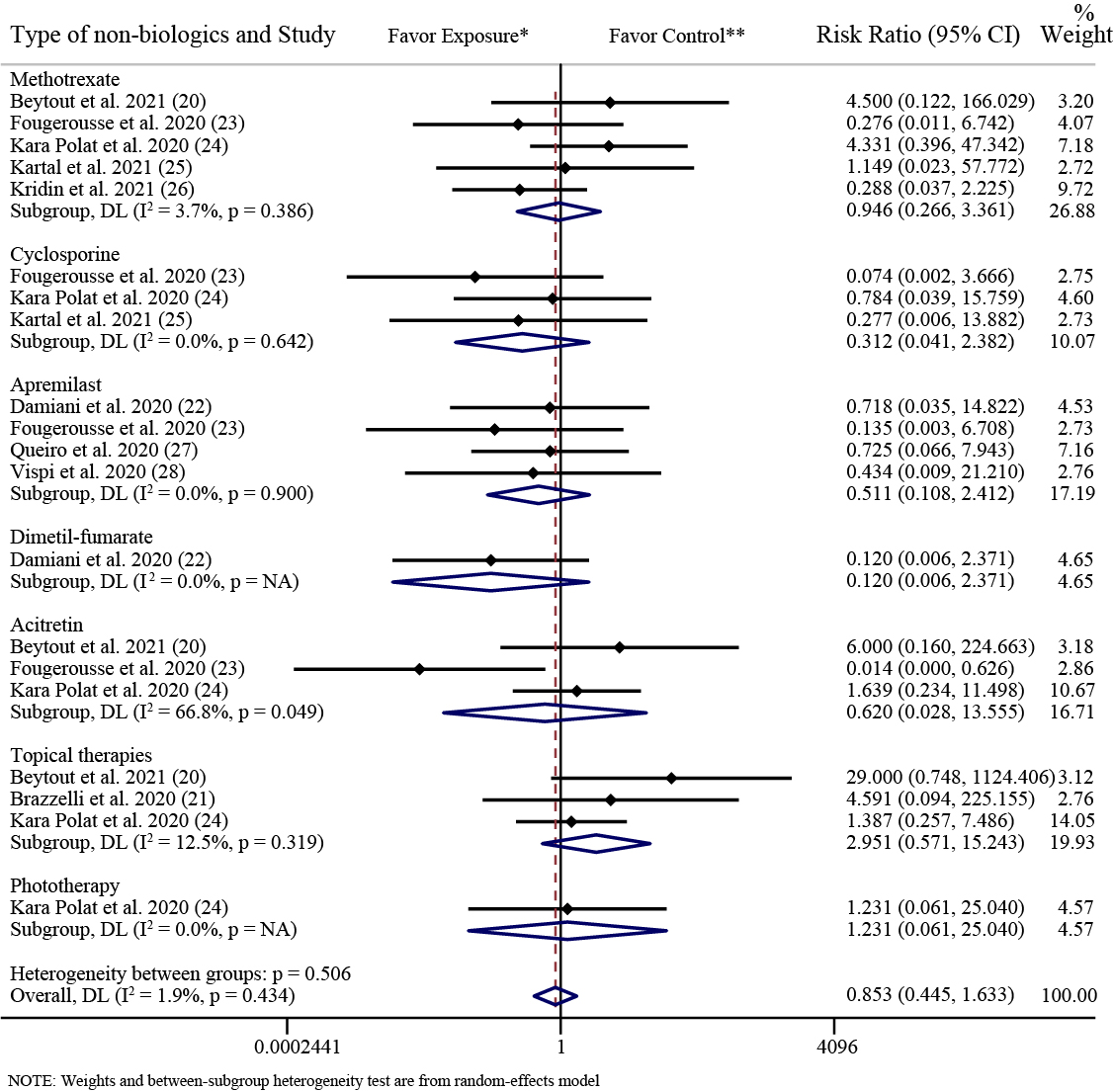


**Supplementary Figure 2**. Forest plot of risk ratios and 95% CIs of the COVID-19 hospitalization rate in comparative analyses (grouped by the specific types of non-biologics). *CI*, confidence interval; *COVID-19*, coronavirus disease 2019; *DL*, DerSimonian-Laird estimate of tau^2^; *NA*, not available. ^*^Exposure represents patients with psoriasis treated with IL-17 inhibitors. ^**^Control represents patients with psoriasis treated with non-biologics.


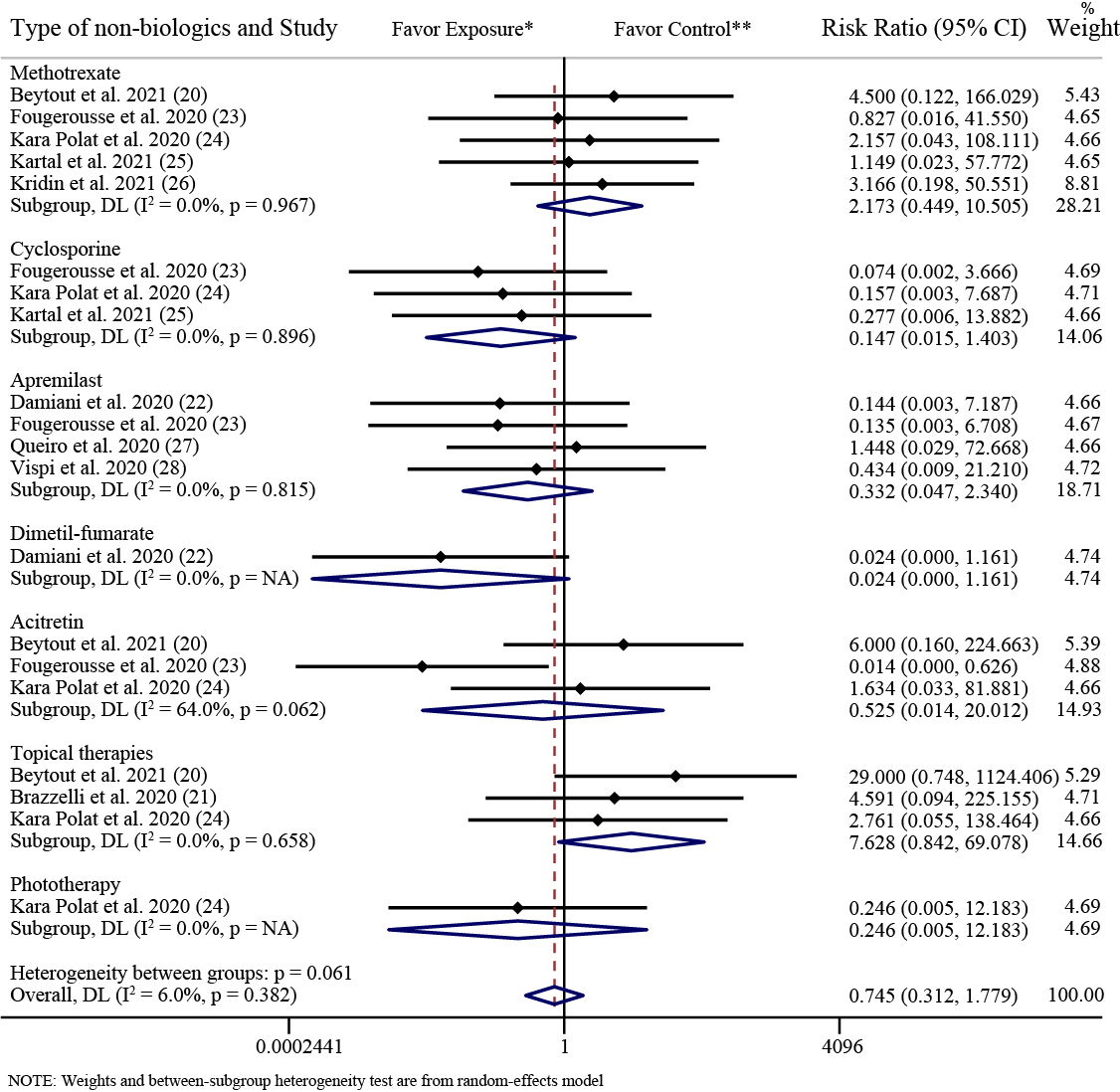


**Supplementary Figure 3**. Forest plot of risk ratios and 95% CIs of the COVID-19 mortality rate in comparative analyses (grouped by the specific types of non-biologics). *CI*, confidence interval; *COVID-19*, coronavirus disease 2019; *DL*, DerSimonian-Laird estimate of tau^2^; *NA*, not available. ^*^Exposure represents patients with psoriasis treated with IL-17 inhibitors. ^**^Control represents patients with psoriasis treated with non-biologics.


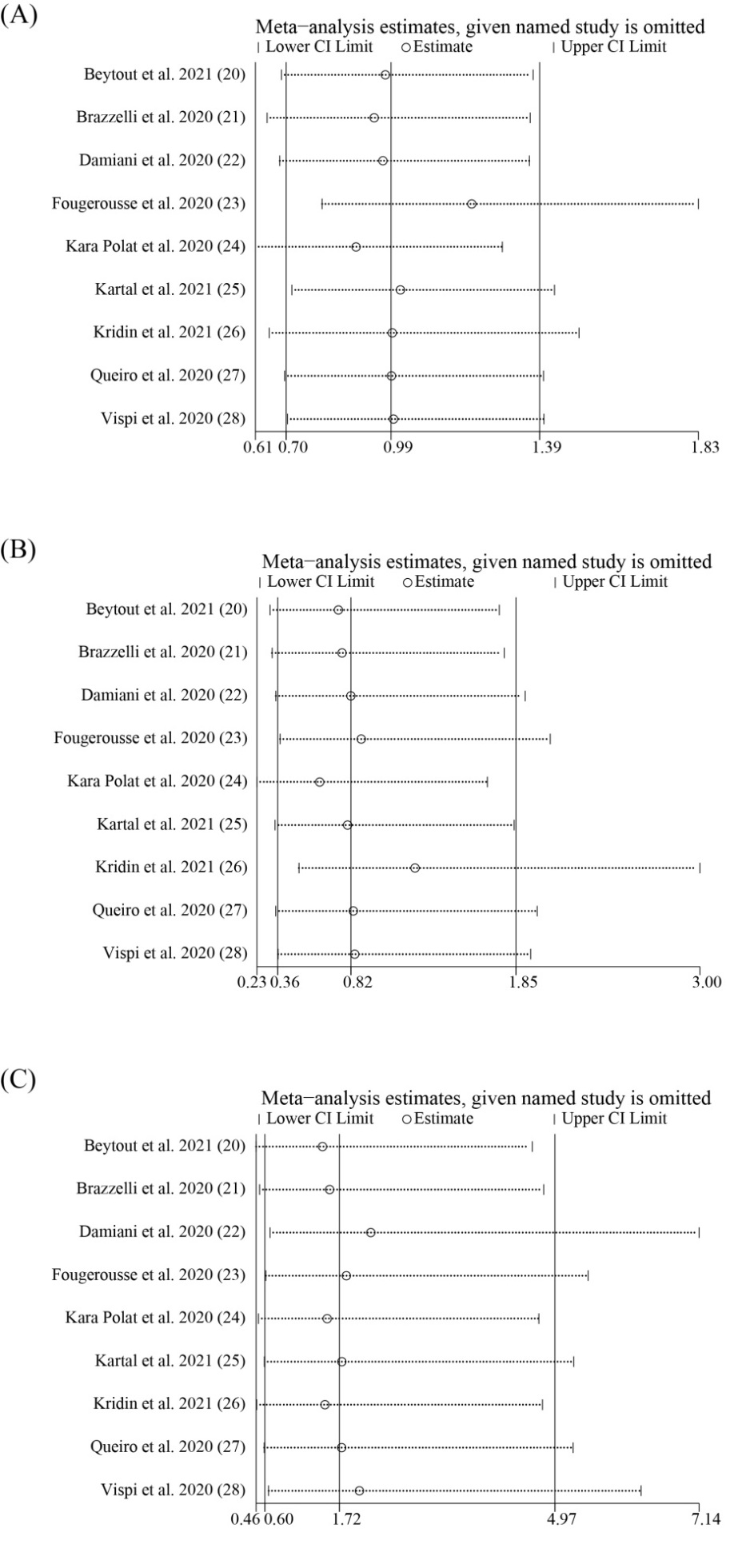


**Supplementary Figure 4.** Sensitivity analysis of the association between IL-17 inhibitors and COVID-19. The influence of individual studies on the overall RR is shown. The middle vertical axis indicates the overall RR, and the two vertical axes indicate the 95% CIs. The hollow circles represent the pooled RR when the remaining study is omitted from the meta-analysis. The two ends of each broken line represent the 95% CI. **(A)** Sensitivity analysis of infection rate of SARS-CoV-2; **(B)** Sensitivity analysis of hospitalization rate of COVID-19; **(C)** Sensitivity analysis of mortality rate of COVID-19. *CI*, confidence interval; *COVID-19*, coronavirus disease 2019; *IL-17*, interleukin-17; *RR*, risk ratio; *SARS-CoV-2*, severe acute respiratory syndrome coronavirus 2.


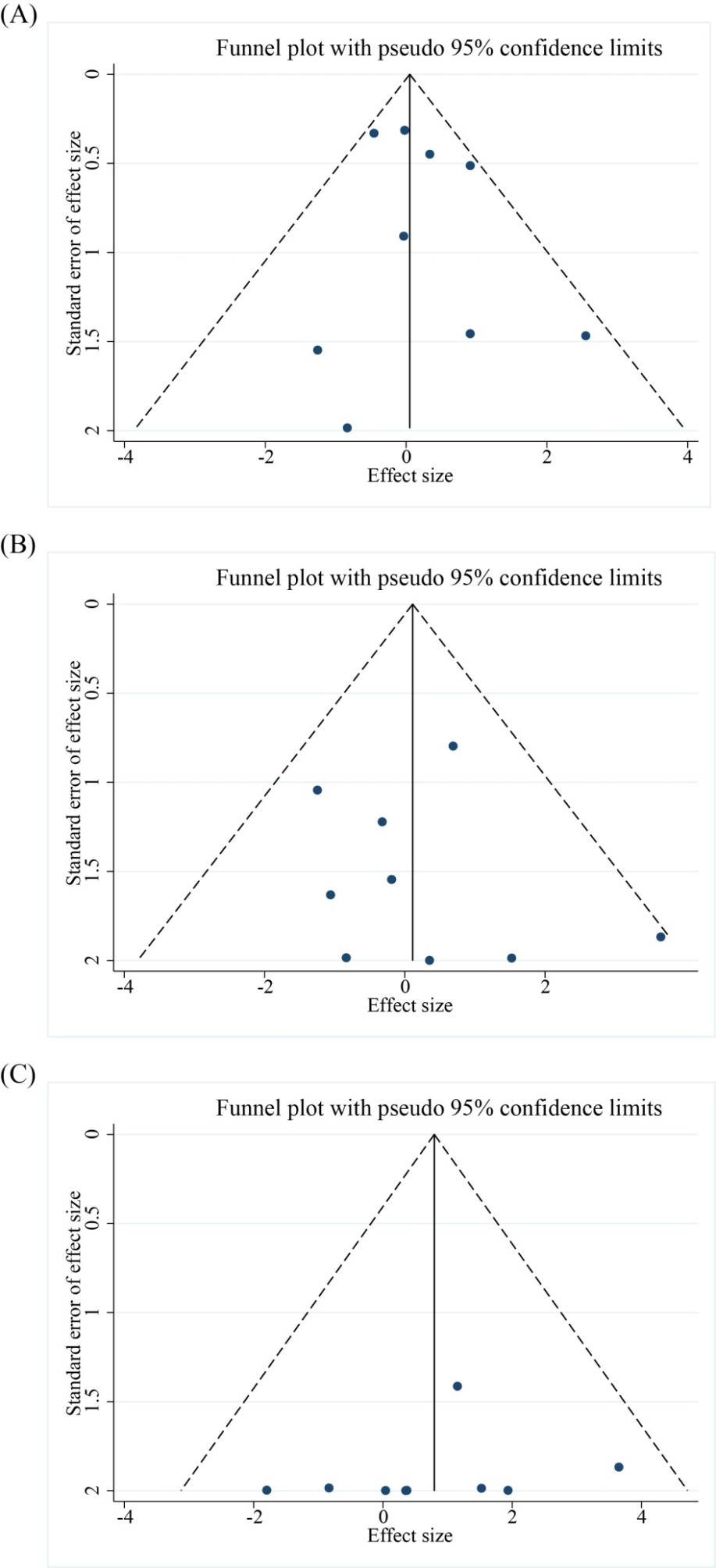


**Supplementary Figure 5.** Funnel plots. **(A)** Funnel plot of infection rate of SARS-CoV-2; **(B)** Funnel plot of hospitalization rate of COVID-19; **(C)** Funnel plot of mortality rate of COVID-19. *COVID-19*, coronavirus disease 2019; *SARS-CoV-2*, severe acute respiratory syndrome coronavirus 2.


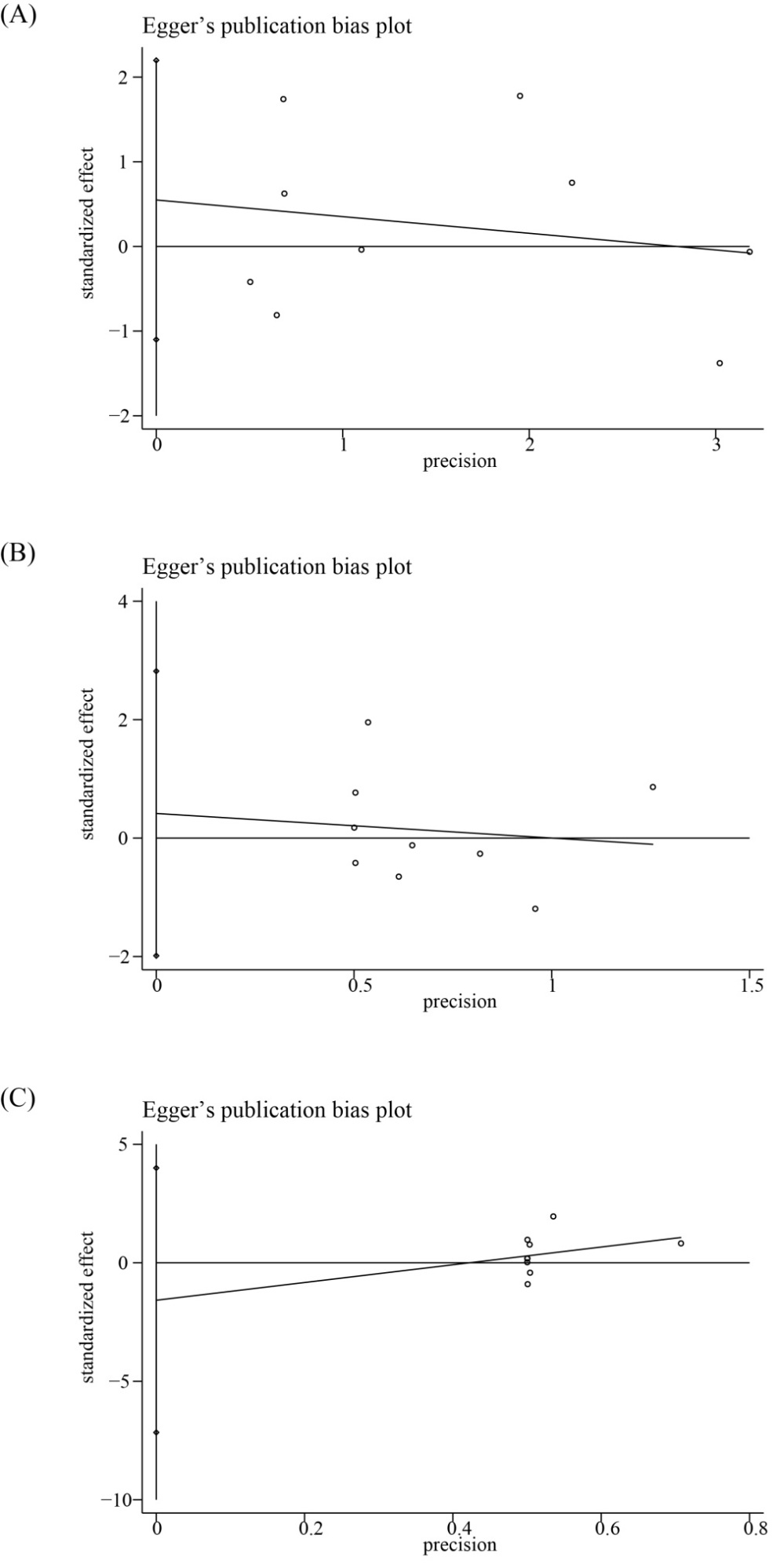


**Supplementary Figure 6.** Egger’s test for publication bias of included studies for COVID-19 outcomes. **(A)** Egger’s test for publication bias of 9 included studies for SARS-CoV-2 infection rate; **(B)** Egger’s test for publication bias of 9 included studies for COVID-19 hospitalization rate; **(C)** Egger’s test for publication bias of 9 included studies for COVID-19 mortality rate. *COVID-19*, coronavirus disease 2019; *SARS-CoV-2*, severe acute respiratory syndrome coronavirus 2.


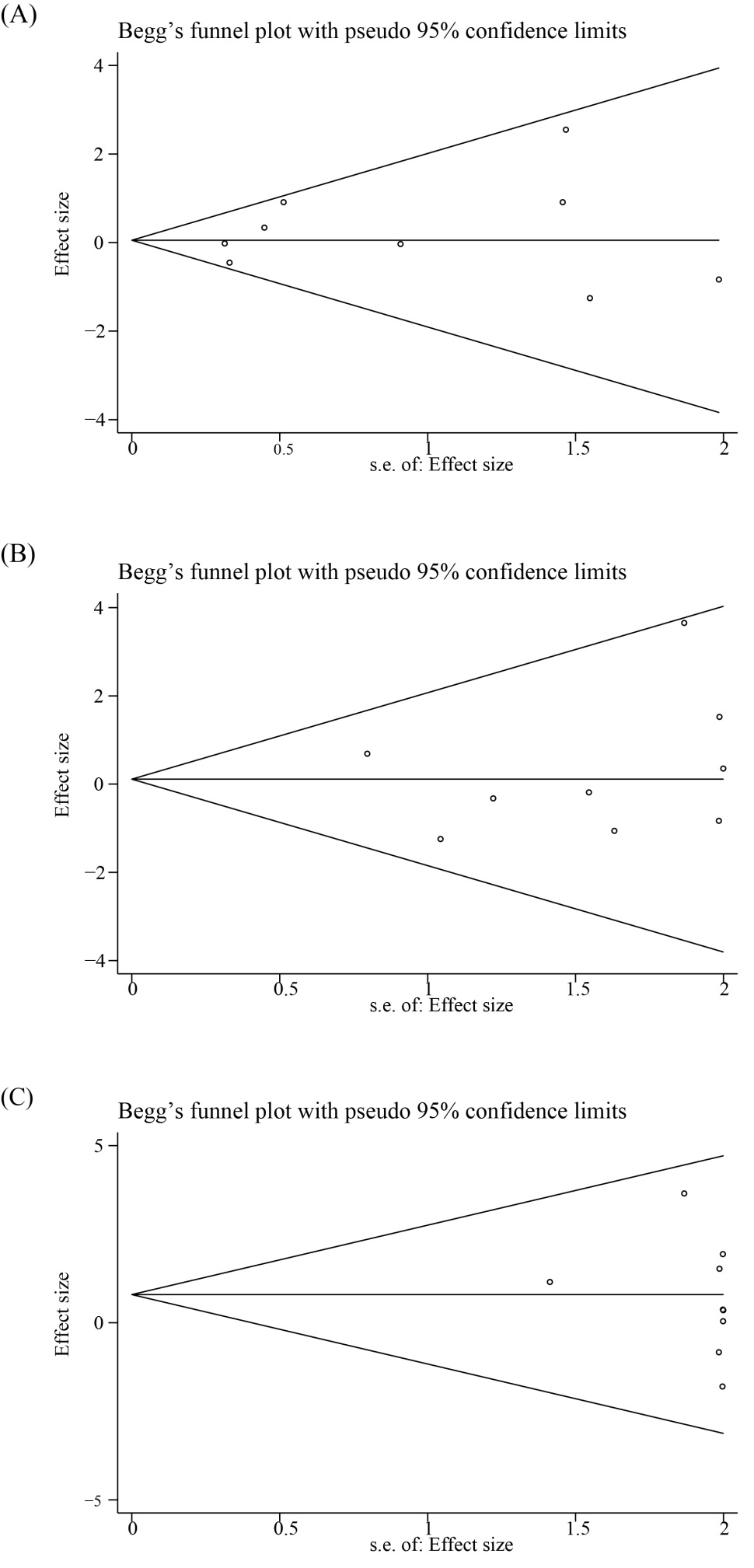


**Supplementary Figure 7.** Begg’s test for publication bias of included studies for COVID-19 outcomes. **(A)** Begg’s test for publication bias of 9 included studies for SARS-CoV-2 infection rate; **(B)** Begg’s test for publication bias of 9 included studies for COVID-19 hospitalization rate; **(C)** Begg’s test for publication bias of 9 included studies for COVID-19 mortality rate. *COVID-19*, coronavirus disease 2019; *SARS-CoV-2*, severe acute respiratory syndrome coronavirus 2.
